# Elemental Composition of Denim Samples via Conductive Heating-Assisted Decomposition with Low Acid Consumption and ICP-OES Detection

**DOI:** 10.1021/acsomega.5c04091

**Published:** 2025-09-02

**Authors:** Amanda Laís Barbosa, Iago José Santos da Silva, Briyitte Sofia Salazar Torres, Ana Paula Silveira Paim

**Affiliations:** a Centro de Ciências Exatas e da Natureza, Departamento de Química Fundamental, Universidade Federal de Pernambuco, Avenida Jornalista Aníbal Fernandes, s/n, Cidade Universitária, Recife, PE, CEP 50740-560, Brazil; b Departamento de Engenharia Civil e Ambiental, 28116 Universidade Federal de Pernambuco, Rua Acadêmico Hélio Ramos, s/n, Cidade Universitária, Recife, PE, CEP 50740-467, Brazil

## Abstract

This study aimed to determine the elemental composition (Al, Co, Cu, Mg, Mn, Ni, and Zn) of denim garments with varying compositions. An acid digestion method was developed using a 2^3^ full factorial design to optimize sample preparation conditions, using an open-system digester block. Among 29 denim samples, 27 were successfully digested, exhibiting residual carbon and acidity below 2% and 10%, respectively. Elemental analysis was conducted using ICP OES, with validation through recovery tests (75–125%) and a certified reference material. No significant differences were found between the determined and certified values, as confirmed by a *t* test. DLS analysis revealed a particle size distribution of less than 1 μm in the digested samples. The method proved to be cost-effective, also reducing acid use by up to 80% compared to previous works. Additionally, the method attained an analytical Eco-Scale score of 83, thereby qualifying as an excellent example of green analytical methodology. While Mg showed the highest concentration (322.2 mg kg^–1^), the elements Co (0.34 mg kg^–1^) and Ni (0.67 mg kg^–1^) exhibited the lowest concentrations. Cu and Mn were found in concentrations higher than recommended levels (50 mg kg^–1^ for Cu and 90 mg kg^–1^ for Mn), with ranges of 0.07 to 89.3 mg kg^–1^ Cu and 1.4 to 608.0 mg kg^–1^ Mn. On the other hand, Ni concentrations were close to the established limit (1 mg kg^–1^ Ni), ranging from 0.57 to 1.0 mg kg^–1^. These findings underscore the need for additional research on denim to identify toxic elements and their associated health risks.

## Graphical abstract

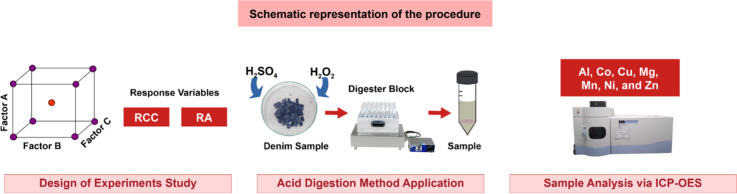

## 1 Introduction

The garment production sector is one of the most dynamic in the global economy, continually launching new products and services. Since the beginning of the century, this sector has experienced substantial growth due to the consumption model known as *fast fashion* — a marketing phenomenon that refers to the increasing supply of garments in parallel with a decrease in their useful lifespan.^1^

It also raises interest in the quality of these products and the impacts associated with their use, especially denim clothes, which are widely used. Brazilian denim production is a global benchmark, with the market generating approximately 4.5 billion US dollars annually.^2^

Cotton fiber is the main component of denim. From the cultivation of cotton, several treatment operations are involved to achieve the desired quality of the final product. In the chemical treatment of fibers and fabrics, the following substances are commonly used: bleaches, oxidizing agents, mordants, dyes and pigments, waterproofing agents, and antifungal agents.^3–5^

Specific chemical techniques, particularly those involving dyeing and pigmentation, can introduce metallic elements – such as aluminum (Al), cobalt (Co), copper (Cu), magnesium (Mg), manganese (Mn), nickel (Ni), and zinc (Zn) – into fabrics. While these elements take part in essential physiological processes, exposure to certain concentration levels may pose risks to human health.

When considering dermal exposure, cobalt and nickel rank among the most common sensitizing agents and are frequently associated with cases of allergic contact dermatitis. This condition is characterized by erythema, pruritus, vesiculation, and in severe cases, exudative lesions. Nickel sensitization is primarily attributed to its release from consumer products that maintain direct and prolonged contact with the skin. Similarly, cobalt exposure may result in cutaneous inflammation and has been linked to both cardiovascular and respiratory dysfunction upon systemic absorption.^6–8^

Copper, widely present in everyday items, may also induce irritation or allergic reactions when it is ionized through prolonged skin contact. Although metallic copper is typically inert, electrochemical reactions at the skin surface can facilitate the release of ions, particularly in the presence of moisture or exudates. Manganese, although less commonly associated with dermal effects, can accumulate in tissues following excessive exposure, primarily through inhalation or ingestion, and has been linked to neurological, reproductive, and respiratory disturbances.^9–11^

Because metallic elements are widely used in textile industry processes and exhibit the capacity to cause health issues, it is critical to understand their presence on clothing in order to assess risks from prolonged exposure.^12^ Dermal contact, particularly long-term or repeated exposure from consumer products, is a significant yet often overlooked absorption pathway.

In the scientific literature, there are reports on the migration of metals from garments to the skin of users.^13–17^ Considering the skin’s daily exposure to clothing, it is necessary to monitor the concentration of metals in textile matrices.

The analytical assessment of chemical elements stands out as a tool for monitoring the occurrence of harmful substances in textile matrices. Inductively Coupled Plasma Optical Emission Spectrometry (ICP OES) is among the most commonly used techniques, as it allows the determination of many elements, standing out for its low detection limits and relatively interference-free detection capacity.^18^

Ghosh et al. (2013)^18^ highlighted advantages of Inductively Coupled Plasma compared to other excitation methods: the high temperatures achieved by the plasma (up to 10,000 K) are responsible for the accuracy and reproducibility observed for a variety of elements in different matrices, characterizing the atomization, ionization, and excitation processes in ICP as less susceptible to interferences.

Several of the available instrumental methods require that the analyte be free in a simple inorganic form, in a liquid and homogeneous matrix. Thus, in chemical analysis, it is often necessary to use sample preparation, which, preceding the determination step, aims to adapt the sample to the analytical method.^19^ Although microwave radiation-assisted digestion is frequently mentioned in the literature on sample preparation, the cost of purchasing, maintaining, and replacing microwave oven parts remains a significant issue, making equipment and its maintenance unaffordable to many laboratories.^20^

From this perspective, there are alternative paths, including open system methods. In addition to aiming for milder conditions and obtaining better quality digestates, these methods foresee the use of lower-cost resources. Among the equipment used in open-system sample preparation, digester blocks are the primary representatives, standing out for their ease of operation, as well as the cost of acquisition, maintenance, replacement of parts, and the analytical frequency. There is also a tendency to combine heating with condensation and reflux systems. Some authors note that the use of these resources yields equally satisfactory results, in terms of merit figures, when compared to closed systems.^21–23^

A powerful ally for the development of sample preparation methods is the concept of Design of Experiments, which provides tools based on response variables to describe the reaction system quantitatively. To develop efficient sample preparation methods for ICP OES analysis, residual acidity (RA) and residual carbon content (RCC) of digested samples are often indicated as the best response variables, as they relate to the amount of acid and organic matter contained in these aliquots. Some authors have established ideal limits for RA and RCC of 10% (v/v) and 12% (w/w), respectively.^24^

Additionally, the particle size distribution is a crucial parameter for describing digested samples and their respective preparation methods. In ICP OES, the presence of particulate matter in digested samples can affect the quality of determinations, influencing transport phenomena and plasma processes. On this basis, the literature establishes a limit of 5 μm for the particle size distribution in digested samples.^25,26^ Thus, it is ensured that the digested samples have transport properties similar to the solutions used in calibration.

The literature on the preparation of textile samples for an analysis of their constituent elements is scarce; a literature review found only one article focusing on denim samples.^13^ To our knowledge, no previous work has proposed diversifying the preparation of denim samples through the use of lower-cost equipment, such as the open-system digester block.

Considering the aforementioned points, this work contributed to the development and validation of an analytical method for determining Al, Co, Cu, Mg, Mn, Ni, and Zn in denim, utilizing a digester block as a heating source. This analytical approach had as its primary purpose the reduction of costs (instrumentation and consumables) associated with sample preparation for elemental determination, including the proposal of a greener procedure to minimize excessive waste generation and reduce standard deviation in analytical measurements.

## 2 Materials and Methods

### 2.1 Instrumentation

To digest the denim samples, a digester block (TE-040/25, Tecnal Equipamentos Científicos, Brazil) with a maximum operating temperature of 450 °C was used, equipped with 15 borosilicate glass tubes (75 mL). The tubes were capped with sterilized penicillin vials (volume: 8 mL; height: 47 mm; diameter: 23 mm) to prevent losses due to volatilization of the acid solution (Figure 1).

**Figure 1:**
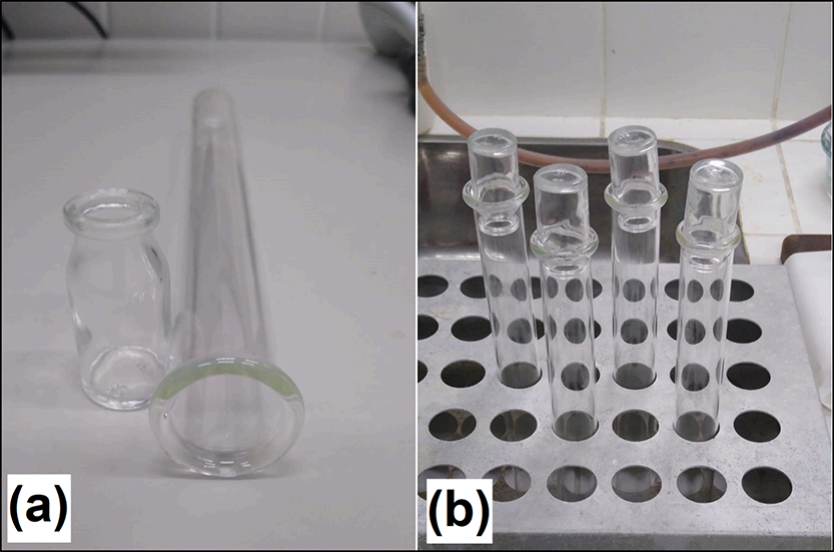
(a) Borosilicate glass tubes (fitted with penicillin vials) used as reaction system flasks. (b) Digestion block with glass tubes.

The Original Carbon Content (OCC) was determined by UV–vis absorption spectra to 620 nm, recorded using a molecular absorption spectrophotometer (Agilent 8453, Agilent Technologies, USA), equipped with quartz cuvettes (1.0 cm) and deuterium and tungsten lamps.

To determine the elements, an ICP OES (Model Optima 7000 DV, PerkinElmer, USA) was used. The following operational parameters were applied: cross-flow nebulizer, Scott spray chamber, radiofrequency power of 1300 W, plasma gas flow rate of 15 L min^–1^, auxiliary gas flow rate of 0.2 L min^–1^, nebulizer gas flow rate of 0.8 L min^–1^, sample flow rate of 1.0 mL min^–1^, flush time of 8 s, delay time of 20 s, and read time of 27 s. The following emission lines (nm) were monitored in the axial view: Al 396.153 (I); C 193.030 (I); Co 228.616 (II); Cr 267.716 (II); Cu 327.393 (I); Fe 238.204 (II); Mg 285.213 (I); Mn 257.610 (II); Ni 231.604 (II); and Zn 213.857 (I). With “I” for atomic lines and “II” for ionic lines.

Dynamic Light Scattering (DLS) analysis was performed using a size particle analyzer (Zetasizer Nano ZS90, Malvern Panalytical, UK) and an ultrasonic bath (Easy 20 H, Elma, Germany) for sample homogenization.

### 2.2 Reagents and Standard Solutions

All solutions were prepared from high-purity analytical reagents and ultrapure water with a specific resistivity of 18.2 MΩ cm (Direct-Q 3 UV, Merck Millipore, USA). Before use, all glassware, tips, and bottles used for the experiments were decontaminated in a 10% HNO_3_ solution for 24 h and were subsequently rinsed with ultrapure water.

To test the sample decomposition, three different reagents were used: 98% H_2_SO_4_, 65% HNO_3_ (both from Merck, Germany), and 30% H_2_O_2_ (Dinâmica Química Contemporânea, Brazil).

The RA was determined through an acid–base titration. For this, a solution of sodium hydroxide (0.1901 mol L^–1^) was prepared and standardized with 0.05 mol L^–1^ potassium hydrogen phthalate. An alcoholic solution of phenolphthalein (1%) was used as the indicator.

The RCC was determined through two independent measurements: the original carbon content (OCC), which refers to the carbon concentration in denim samples, and the dissolved carbon content (DCC), referring to the carbon concentration in the digested samples. RCC is equivalent to the quotient between OCC and DCC.^24^

To determine the DCC in digested samples via ICP OES, a stock solution of d-glucose (Synth, Brazil) containing 50,000 mg L^–1^ was prepared, which is equivalent to 5% carbon. From this stock, standard calibration solutions were prepared in the range of 100 to 4000 mg L^–1^ of carbon.

The OCC was determined using a spectrophotometric method. Stock solutions of potassium dichromate (Dinâmica Química Contemporânea, Brazil) and potassium hydrogen phthalate (Merck, Germany) were prepared in concentrations of 50 mg L^–1^ and 10000 mg L^–1^, respectively.

Standard solutions were prepared from a 1000 mg L^–1^ multielement standard (Inorganic Ventures, USA) containing the following elements: Al, Co, Cr, Cu, Fe, Mg, Mn, Ni, and Zn. The analytical curve was prepared from dilutions of the stock solution, with concentrations ranging from 0.1 to 8.0 mg L^–1^. To increase stability, these solutions were prepared in 5% HNO3, thereby avoiding the precipitation and adsorption of the analytes.

### 2.3 Samples

A total of 29 (twenty-nine) denim samples with variable compositions (Cotton - CO, Elastane - EA, and Polyester - PL) were acquired from local stores in the municipalities of Recife and Caruaru (PE, Brazil). To ensure representative sampling, each sample was taken from different places. The patches were then cut into smaller pieces (approximately 6 × 6 mm), which were identified and stored in ziplock plastic bags (6 × 5 cm) until sample preparation. For cutting with less possibility of contamination, scissors coated with titanium were used. All experiments were conducted using samples cut and stored as described in Table S1 (Supporting Information).

### 2.4 Sample Preparation

#### 2.4.1 Sample Preparation in the Digester Block

All samples were treated with an acidic solution and heated in the digester block. Each experiment was conducted by adding the solution and the corresponding sample mass to the test tube, which was then sealed and proceeded with the selected heating program (described in Section 2.4.2). All the preparations were carried out at 120 °C, considering the boiling point of acid solutions. All digested samples were diluted to a final volume of 20 mL. Later, based on the experimental design, the dilution factor was adjusted, and the final volume was changed to 30 mL.

#### 2.4.2 Preliminary Tests

Initially, preliminary tests were conducted to evaluate the efficiency of various acid solutions in digesting denim samples. The conditions used in those experiments were selected according to previous works. The original acid solution volumes were adjusted to a mass of 200 mg of sample. Sample T7, due to its high polyester content, was considered the most difficult to digest and was therefore selected for preliminary tests.

For the decomposition of textile samples, some studies suggest using HNO_3_ and H_2_O_2_ as auxiliary oxidizing agents.^13,14,27^ Taking these references into account, the acid mixture was evaluated according to the following conditions: 8 mL of HNO_3_ (25%, 50%, and 65%), 2 mL of 30% H_2_O_2_, heated with the sample for 2 h at 120 °C.

Other authors suggest the use of an acidic mixture of HNO_3_ and H_2_SO_4_.^28,29^ Thus, this mixture was evaluated under the following conditions: 50% HNO_3_ in variable volumes (10, 6, and 2 mL) and 50% H_2_SO_4_ in variable volumes as well (2, 6, and 10 mL), resulting in a final volume of 12 mL.

For the decomposition of recycled polyester, some authors suggest using H_2_SO_4_ and H_2_O_2_.^30^ This mixture was evaluated under the following conditions: 4 mL of H_2_SO_4_ (98, 74, and 49%), 4 mL of 30% H_2_O_2_, heated with the sample for 2 h at 120 °C.

Other publications suggest that H_2_SO_4_ must be used in its concentrated form to decompose the samples effectively.^23^ Therefore, as an additional modification, the reaction solution was added in two steps: (I) 2 mL of 98% H_2_SO_4_ along with 1 mL of 30% H_2_O_2_ and the sample, heating the system from room temperature to 120 °C; (II) 4 mL of 30% H_2_O_2_, heating the system for 2 h at 120 °C.

#### 2.4.3 Full Factorial Design

Based on the literature and the preliminary tests, the H_2_SO_4_/H_2_O_2_ mixture was selected as the best digestion solution. A complete factorial design (2^3^) with central point and triplicate was carried out, totaling 27 experiments. The objective was to evaluate the influence of three variables on digestion efficiency: heating time (60, 90, and 120 min), volumetric ratio of H_2_SO_4_ to H_2_O_2_ (2, 3, and 4 mL mL^–1^), and volume of 30% H_2_O_2_ (4, 5, and 6 mL). The parameters RCC and RA were chosen as dependent variables (responses) for the experimental design. Based on preliminary tests, a 2^3^ full factorial design was implemented, selecting the sample with code T7 for the reasons previously explained. Thus, if the method obtained were efficient in digesting denim with high polyester content, it would likely be efficient in decomposing simpler types of denim as well.

### 2.5 Assessment of the Response Variables: RA and RCC

The RA was determined through titration with the NaOH standard solution (0.1901 mol L^–1^). Each assay was prepared by diluting aliquots taken from digested samples (250 μL) to a final volume of 4 mL with ultrapure water. All assays were carried out in triplicate (n = 3).

The RCC values were determined through two independent measurements: the DCC of the digested samples and the OCC of the denim samples. The DCC was assessed through ICP OES analysis (carbon emission line: 193.030 nm); the OCC was evaluated using the spectrometry method.^31^

### 2.6 Method Validation

Validation is a crucial tool for developing analytical methods, essential for obtaining reliable results. The method was validated by obtaining the following performance parameters: linearity (correlation coefficient), limit of detection (LOD), limit of quantification (LOQ), precision (relative standard deviation – %RSD), and accuracy. The limits of detection and quantification were calculated using the blank reading of the analytical curve, as follows: LOD = 3.3 × S_blank_/b and LOQ = 10 × S_blank_/b (at a 99% confidence level). Where “S_blank_” is the standard deviation of the blank analytical signal (n = 9) and “b” is the slope of the analytical curve. Accuracy tests were performed employing the RM Agro E1001a *Brachiaria Brizantha* as certified reference material (CRM) (Table S2 - Supporting Information).^32^

All nine elements were checked for precision and accuracy through recovery tests. These tests were performed at two concentrations: 2 and 4 mg L^–1^ for Mg; and 0.4 and 0.8 mg L^–1^ for the other elements. The levels were selected based on the concentrations obtained in the samples, considering the analytical curve range. A total of four different samples were used (T3, T7, T24, and T29) to verify denim samples with varying fiber compositions (Table S1 – Supporting Information).

### 2.7 Dynamic Light Scattering

The DLS analysis was performed on digested samples to evaluate the particle size distribution in these solutions. Three digested samples were randomly chosen, referring to denim clothes coded as T4, T7, and T11 (Table S1 - Supporting Information). For homogenization, the solutions were kept in an ultrasonic bath for 20 min, and then, aliquots of 2 mL were taken for analysis. All experiments were carried out in triplicate.

## 3 Results and Discussion

### 3.1 Effect of Acid Solution

Most published methods for sample preparation in elemental analysis utilize microwave radiation as a heating source.^13–16^ Therefore, using a digester block, preliminary tests were carried out to evaluate the efficiency of three different acid solutions in decomposing denim samples. Table S3 (Supporting Information) summarizes the main results obtained from the preliminary tests.

Visually, out of the preliminary tests performed, most exhibited partially decomposed material with solid residues dispersed in a solution of yellow coloration, indicating incomplete sample digestion (Figure 2, pictures 1 to 4). On the other hand, the tests performed with the third acid mixture (H_2_SO_4_/H_2_O_2_) showed no noticeable solid residues or coloration (Figure 2, picture 5). Thus, the mixture of H_2_SO_4_ and H_2_O_2_ was selected for the following stages of the study.

**Figure 2:**
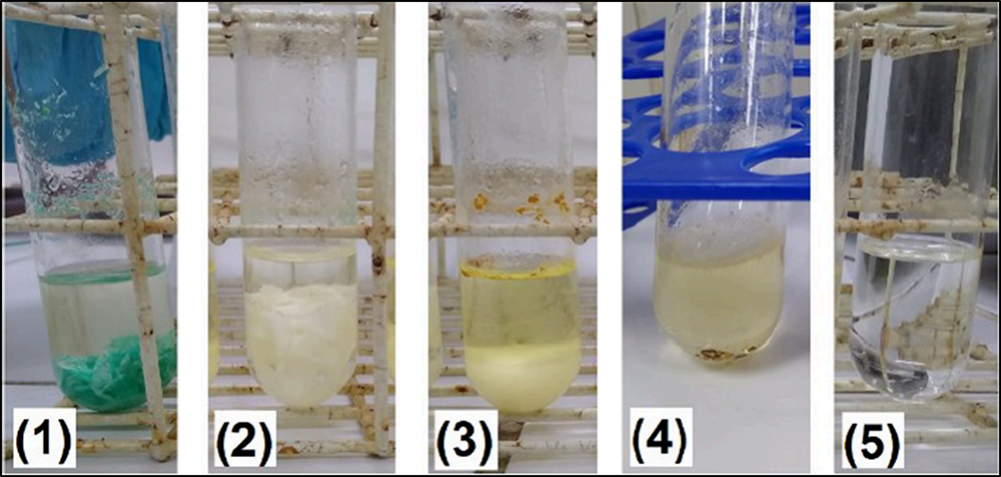
Visual aspect of digested samples: whole pieces of the sample (1); partially decomposed pieces (2); particulate material in large quantity (3); particulate material in small quantity (4); absence of particulate material (5).

The mixture H_2_SO_4_/H_2_O_2_, known as a “piranha solution” has a high oxidizing potential and is commonly used in laboratory procedures for removing organic residues from surfaces.^33^ The effectiveness of this mixture lies in the synergistic action of sulfuric acid and hydrogen peroxide: (I) the interaction between H_2_SO_4_ and H_2_O_2_ generates oxygen radical species, and (II) concentrated H_2_SO_4_ promotes the carbonization of the sample. These reactive species attack the unsaturated bonds present in the carbonized compounds, leading to the release of CO_2_ as the final product of the decomposition process. In the presence of a sufficient amount of sulfuric acid and hydrogen peroxide, the reaction proceeds until the sample is completely degraded.^34^

### 3.2 Full Factorial Design

Preliminary tests showed that the use of concentrated sulfuric acid is necessary for a complete carbonization of the samples. Additionally, the combined action of acid and peroxide promotes decomposition. Considering these aspects, as previously mentioned, it was decided to add the reaction mixture in two steps. Thus, the variable volumetric ratio (Factor A) refers to the quotient between the volumes of H_2_SO_4_ (V_1_) and H_2_O_2_ (V_2_) selected for the first step of sample digestion (Table 1).

**Table 1:** Matrix of Complete Factorial Design (2^3^) for Denim Digestion and Respective Responses in Residual Carbon Content (RCC) and Residual Acidity (RA)

factors				(−)	(0)	(+)	reaction steps	
A	volumetric ratio (mL mL^–1^)^a^			2	3	4	(I) sample (200 mg) + H_2_SO_4_ (*V*_1_) + H_2_O_2_ (*V*_2_)	
B	volume of 30% H_2_O_2_ (mL)^b^			4	5	6	(II) H_2_O_2_ (V_3_), 120 °C	
C	heating time (min)			60	90	120		
experiment	A	B	C	*V*_1_ (mL)	*V*_2_ (mL)	*V*_3_ (mL)	RCC (% w/w)^c^	RA (% v/v)^c^
E1	–	–	–	2.0	1.0	4	2.7 ± 0.2	15.4 ± 0.2
E2	+	–	–	2.4	0.6	4	3.2 ± 0.5	17.5 ± 0.3
E3	–	+	–	2.0	1.0	6	2.5 ± 0.7	15.5 ± 0.6
E4	+	+	–	2.4	0.6	6	2.3 ± 0.6	18.0 ± 0.3
E5	–	–	+	2.0	1.0	4	2.4 ± 0.8	15.3 ± 0.5
E6	+	–	+	2.4	0.6	4	2.4 ± 0.8	17.6 ± 1.0
E7	–	+	+	2.0	1.0	6	1.7 ± 0.3	15.3 ± 0.5
E8	+	+	+	2.4	0.6	6	1.7 ± 0.4	18.9 ± 0.8
E9	0	0	0	2.25	0.75	5	1.8 ± 0.3	17.0 ± 0.6

a *V*_1_ + *V*_2_ = 3 mL. Also, *V*_1_/*V*_2_ = volumetric ratio of H_2_SO_4_ to H_2_O_2_ (mL mL^–1^), and this ratio refers only to step I of digestion.

b The volume of 30% H_2_O_2_ (Factor B and V_3_) refers to step II of digestion.

c Mean value ± standard deviation (*n* = 3).

A **2** ^3^ experimental design was elaborated to evaluate the influence of different variables on the RA and RCC responses, aiming to meet the limits established in literature and also defining milder digestion conditions. Based on the preliminary test results, three factors were selected to build a full factorial design: volumetric ratio (A), volume of 30% H_2_O_2_ (B), and heating time (C), as indicated in Table 1.

The RA and RCC results obtained in each experiment are presented in Figure 3. As shown, some RA values exceeded the maximum threshold recommended in the literature, defined as ≤ 10% (v/v).^24^ To address this issue, the dilution factor was adjusted, and the final volume was increased from 20 to 30 mL, which effectively reduced RA values to below the established limit. In contrast, RCC values remained consistently low across all evaluated conditions, ensuring concentrations below 12% (w/w). These results demonstrate the efficiency of the proposed method for digesting denim samples, by established quality criteria.

**Figure 3:**
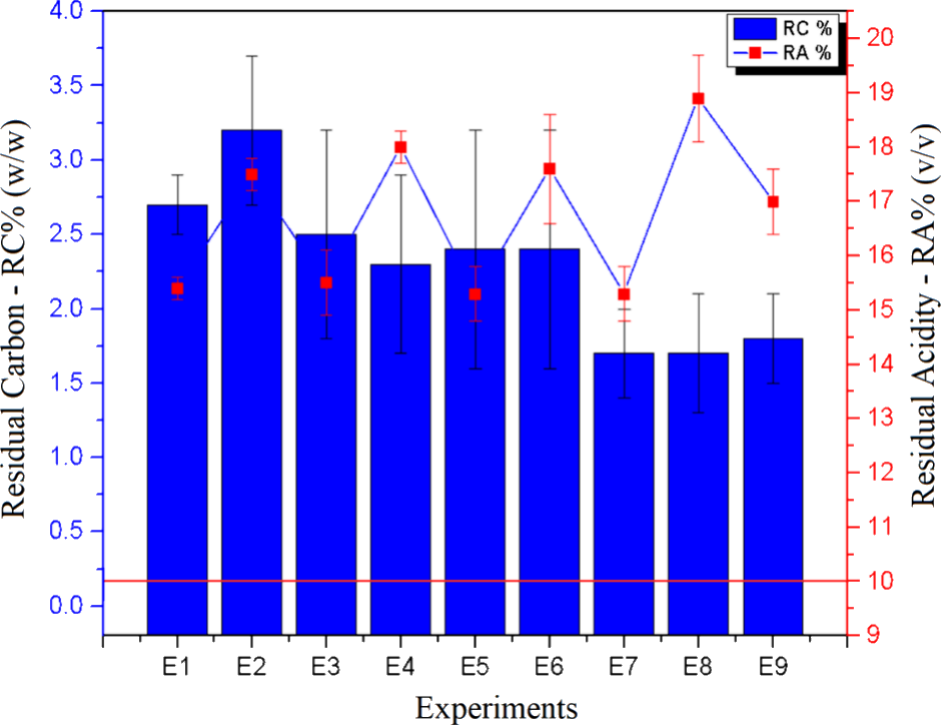
Sample digestion conditions: (I) sample (200 mg) + H_2_SO_4_ (*V*_1_) + H_2_O_2_ (*V*_2_); (II) H_2_O_2_ (*V*_3_), 120 °C. Bars (left axis) and lines (right axis) represent the RCC and RA values, respectively. Error bars show the standard deviation (*n* = 3). The horizontal red line represents the acceptable levels of RA for ICP OES.

The RCC values ranged from 1.7% to 3.2%, with experiments E7–E9 yielding the lowest concentrations. Notably, among these, experiment E7 also exhibited the lowest RA, with a value of 15.3%. In addition to elemental analysis, this study also aimed to incorporate green chemistry principles by selecting conditions that comply with literature-recommended limits without compromising analytical performance.

Accordingly, given that all RCC values remained below the 12% (w/w) threshold, the conditions of *Experiment E1* were selected for the digestion of all samples. This experiment represents the mildest conditions within the experimental design, employing minimal reagent volumes: 2 mL of H_2_SO_4_ and 1 mL of H_2_O_2_ in the first stage, followed by 4 mL of H_2_O_2_ in the second stage, with a total heating time of 60 min (Table S4 - Supporting Information). These conditions proved suitable for subsequent analysis by ICP OES.

A brief comparison with previous studies (Table 2) demonstrates that the proposed method was effective for textile sample digestion, employing relatively small volumes of acidic solution in proportion to the sample mass, specifically a ratio of 100 mg of denim per milliliter of sulfuric acid (100 mg mL^–1^).

**Table 2:** Digestion Methods Reported in the Literature that Employed Acid Solutions for Decomposing Different Textile Samples

sample	reagents	ratio (mg mL^–1^)^a^	RCC (% w/w)^b^	RA (% v/v)^b^	reference
raw textile fibers	500 mg of sample; 8 mL of 7 mol L^–1^ HNO_3_	62.5			^27^
polyamide sports clothes	250 mg of sample; 2 mL of 15.8 mol L^–1^ HNO_3_	125	0.2	4	^14^
garments	500 mg of sample; 10 mL of 7.9 mol L^–1^ HNO_3_	50			^40^
home textiles	300 mg of sample; 8 mL of 15.8 mol L^–1^ HNO_3_; 2 mL of 30% H_2_O_2_	37.5			^41^
denim clothes	300 mg of sample; 5 mL of 15.8 mol L^–1^ HNO_3_; 2 mL of 30% H_2_O_2_	60			^13^
recycled polyester fiber	100 mg of sample; 5 mL of 18.4 mol L^–1^ H_2_SO_4_; 5 mL of 30% H_2_O_2_	20			^30^
denim clothes	200 mg of sample; 2 mL of 18.4 mol L^–1^ H_2_SO_4_; 5 mL of 30% H_2_O_2_	100	2.7	10	this work

a Ratio: sample mass (mg) ÷ volume of acid solution (mL).

b Not reported (−).

Compared with the others, the excellent ratio observed for the present method, of 100 mg of sample per 1 mL of acid, was due to the combined action of H_2_SO_4_ and H_2_O_2_, as previously detailed.

### 3.3 Method Validation

The results of validation are shown in Table 3. All elements presented a correlation coefficient (r) greater than 0.995, which allows the method to be considered linear.^35^

**Table 3:** Figures of Merit of the Analytical Method for the Determination of Different Elements in Denim Samples by ICP OES

					RM Agro E1001a Brachiaria brizantha	
element	analytical curves^a^	*r*	LOD (mg kg^–1^)^b^	LOQ (mg kg^–1^)^b^	determined values (mg kg^–1^)^c^	certified values (mg kg^–1^)^d^
Al	*S* = 288,400*C* – 1194.5	0.9988	0.16	0.49		NC
C	*S* = 211.6*C* – 1383.8	0.9999	3.7	11		NC
Co	*S* = 60,510*C* – 233.3	0.9987	0.12	0.35		NC
Cr	*S* = 127,500*C* – 1173.4	0.9999	0.020	0.060	2.7 ± 0.3	3.3 ± 1.7
Cu	*S* = 319,300*C* – 549.6	0.9999	0.080	0.23	3.5 ± 0.1	4.0 ± 0.7
Fe	*S* = 67,443.4*C* + 128.3	0.9984	0.43	1.3	129.7 ± 10.5	91.0 ± 13.0
Mg	*S* = 371,600*C* – 2527.7	0.9984	2.2	6.7	2210 ± 100	2950 ± 440
Mn	*S* = 1,201,000*C* – 3345.1	0.9983	0.020	0.060	59.2 ± 1.2	76.0 ± 18.5
Ni	*S* = 43,370*C* – 249.1	0.9989	0.57	1.7		NC
Zn	*S* = 81,120*C* + 343.8	0.9979	0.16	0.49	9.0 ± 3.6	9.9 ± 1.6

a *S* = analytical signal; *C* = concentration (mg L^–1^).

b In the calculation of LOD and LOQ, the sample mass of denim used in the method (200 mg) and the final volume at which the digested samples were diluted (30 mL) were taken into account. Thus, LOD (mg kg^–1^) = 150 × LOD (mg L^–1^).

c Mean value ± standard deviation (*n* = 3).

d Noncertified (NC).

The elements Al, Co, and Ni were not present on the CRM, and the elements Cr, Cu, Mn, and Zn exhibited concentration values equivalent (at a confidence level of 95%) to the certified ones. For the elements Iron (Fe) and Magnesium (Mg), there was a statistically significant difference between determined and certified concentrations.^36,37^ This result suggests a potential source of contamination among the sample preparation stages: the possible cause for nonvalidation, related to Fe and Mg, could be high blank values.

To validate the method regarding precision and accuracy, addition and recovery tests were carried out.^32,38^ Additions were made at two concentration levels: 2 and 4 mg L^–1^ for Mg; and 0.4 and 0.8 mg L^–1^ for the other elements. According to Table S5 (Supporting Information), most recovery values showed compliance with the limits established by the US EPA (2018),^35^ with recovery values ranging from 75 to 125% for the following elements: Al, Co, Cu, Mg, Mn, Ni, and Zn. Additionally, RSD values of up to 20% were determined, which is within the limits established by the US EPA for ICP OES analysis.

The Fe showed high recovery values for all samples and levels studied, while the elements Cr, Co, and Zn exhibited low recovery values for a considerable number of the tests. The physical interferences caused by mineral acids in ICP OES, as reported in the literature, do not fully account for the behavior observed for Fe in this study.^39^ Contrary to expectations, no significant loss of Fe due to volatilization was detected, despite the use of an open digestion system, which is a condition under which such losses could occur even with a condensation setup in place. Based on these findings, it is reasonable to assume that a potential source of contamination during the sample preparation steps may have contributed to the elevated Fe concentrations observed.

The analytical performance parameters obtained, along with the results from the CRM, confirm that the proposed method is suitable for preparing textile samples for elemental determination by ICP OES (Table 3). Similarly, the results of the addition and recovery tests support the robustness and applicability of the method across various types of denim samples, regardless of the presence of synthetic fibers in their composition (Table S5 - Supporting Information). Upon completion of the validation process, all seven elements evaluated (Al, Co, Cu, Mg, Mn, Ni, and Zn) yielded satisfactory results across all assessed quality criteria.

### 3.4 Particle Size Distribution in Digested Samples

To evaluate the digested samples, particle size distribution was assessed using DLS analysis, which was employed in this study as an effective method to estimate the presence of residual particulate matter. This technique enabled verification of compliance with the literature-established criterion intended to prevent interferences during analyte transport and atomization. The method had previously been applied in the analysis of deodorant and antiperspirant samples.^25^ The digested samples were randomly selected and corresponded to denim fabrics with different fiber compositions: sample T4 (96% CO, 4% EA), sample T7 (82% CO, 16% PL, 2% EA), and sample T11 (77% CO, 20% PL, 3% EA).

The DLS analysis yielded the following results: 523.0 ± 60.0 nm; 568.7 ± 117.2 nm; and 600.6 ± 98.3 nm to sample T4, T7, and T11, respectively. The particle size distribution was evaluated under standard conditions of viscosity (0.8872 cP), refractive index (1.330), temperature (25.0 °C), and measurement position (4.65 nm).

The DLS results confirmed that the digested samples exhibited a particle size distribution suitable for elemental analysis by ICP OES. In all cases, the average particle size remained below the 5 μm threshold established in the literature,^26^ regardless of the fiber composition of the denim samples.

### 3.5 Elemental Determination in Denim Samples

The method developed was applied in the digestion of 29 (twenty-nine) denim samples, and efficiently digested 27 (twenty-seven) of them. Sample digestion was carried out in triplicate, followed by the determination of seven (7) elements — Al, Co, Cu, Mg, Mn, Ni, and Zn — by ICP OES.

The summary of results is shown in Table 4. The quantity “% Detected” refers to the percentage of samples for which the aforementioned element was found in a concentration greater than the LOD. While Mg showed the highest average concentration value (322.2 mg kg^–1^), the elements Co (0.34 mg kg^–1^) and Ni (0.67 mg kg^–1^) exhibited the lowest values. Meanwhile, Co and Ni exhibited concentrations lower than their respective LODs for approximately 30% of samples.

**Table 4:** Elemental Composition of Denim Samples, Expressed in Mean Concentrations (mg kg^–1^), Compared with Values from the Literature of Textile Sample Preparation

			mean concentration (mg kg^–1^)			
element	% detected	concentration range (mg kg^–1^)	this work	Rovira et al.^40^	Rovira et al.^41^	Herrero et al.^13^
Al	100	39.7–145.7	78.6	31.8	14.7	41.2
Co	72	<0.12–0.96	0.34	0.21	0.05	0.04
Cu	97	0.07–89.3	4.9	20.1	32.8	2.2
Mg	100	128.5–752.9	322.2	129.0	142.0	165.0
Mn	100	1.4–608.0	69.0	1.8	0.91	37.6
Ni	66	<0.57–1.0	0.67	<0.05^a^	0.19	1.9
Zn	100	1.2–14.3	7.3	12.1	1.6	4.2

a Values smaller than the LOD.

The composition of elements in denim (and other fabrics) depends on several aspects. Scientific literature reports the most significant ones: fiber composition and chemical treatments involved in the production of these articles. Some authors report that fabrics produced from natural or artificial fibers may have a specific profile for a given set of elements.^13,16,40,41^

For example, Herrero and collaborators (2019)^13^ studying denim clothes, observed differences between garments made from natural and synthetic fibers: their study pointed out that the chemical elements antimony (Sb) and titanium (Ti) were present in greater concentrations in samples with a higher percentage of synthetic fibers in their composition.

The average concentration of metals obtained in our denim samples was compared with the average values reported in the literature (Table 4). The elementary content of the textile samples is quite diverse. Considering multiple sample matrices, open or closed system digestion methods, and different instrumental techniques for elemental determination, our results are very similar to those reported in previous publications, especially the work of Herrero et al. (2019),^13^ which studied denim samples.

In the case of manganese (Mn), the average concentration determined in our study (69.0 mg kg^–^^1^) was higher than most values reported in the literature, except for the study by Herrero et al. (2019), which also focused on denim samples and reported a concentration of 37.6 mg kg^–^^1^. This difference may be attributed to the use of manganese-based compounds in textile finishing processes.^42,43^

Specifically, potassium permanganate (KMnO_4_), a potent oxidizing agent widely employed to produce faded or worn effects on denim garments, can leave residual manganese in the fabric.^44^ The results obtained suggest that the samples analyzed were subjected to this treatment, which would explain the elevated Mn content observed. Given that this metal is not biodegradable^45^ and has been associated with potential dermal effects following prolonged skin contact, it is essential to implement appropriate controls and establish concentration limits for manganese in textile products intended for human use.

Legislation regulating the content of harmful elements in clothing and textile fibers is scarce. Two important references in this field are the Oeko-Tex Standard^46^ and the Global Organic Textile Standard.^47^ The aforementioned are certification systems that provide parameters for the composition of various textile matrices.

Both standards distinguish chemical element concentrations into two categories: extractable and total content, depending on whether the concentration refers to values accessed through migration tests or sample analysis, respectively. In this context, the summarized concentrations in Table 4 refer to the total content of each element in the samples.

Oeko-Tex establishes limits of 25 mg kg^–1^ and 50 mg kg^–1^ for extractable Copper (Cu) in baby clothes and clothes in direct contact with the skin, respectively. For the element Manganese (Mn), these limits are both 90 mg kg^–1^; for the element Nickel (Ni), these limits are both 1 mg kg^–1^.

Taking these limits into account, there is experimental evidence that the elements Cu and Mn might occur in denim samples in concentrations higher than those considered appropriate by standards, referring to the concentration ranges of 0.07 mg kg^–1^ to 89.3 mg kg^–1^ (for Cu), and 1.4 mg kg^–1^ to 608.0 mg kg^–1^ (for Mn). On the other hand, the element Ni exhibited concentration values very close to the established limits, ranging from 0.57 mg kg^–1^ to 1.0 mg kg^–1^.

Since, for some denim samples, the concentrations of Cu and Mn exceeded the limits established for extractable content, further studies are necessary to evaluate the potential migration of these elements to the skin of users.

### 3.6 Analytical Eco-Scale for Assessing the Greenness of the Analytical Method Developed

In addition to the analytical parameters and results that demonstrate the quality of the methodology, particularly in terms of sample preparation and elemental analysis, it is also essential to evaluate the sustainability of the developed approach. To this end, an analytical Eco-Scale proposed in the literature was adopted as a reference to assess the greenness of the method developed in this study.^48^

The semiquantitative approach enables the comparison and selection of greener analytical alternatives by assigning penalty points based on factors such as the type and volume of reagents used, energy consumption, and waste generation. By doing so, the Eco-Scale incorporates environmental considerations aligned with green chemistry principles.

The analytical Eco-Scale is based on the premise that an ideal green analytical method achieves a score of 100. Penalty points are subtracted from this value for each aspect of the analytical procedure that deviates from the principles of green chemistry. The final score categorizes the method’s greenness as follows: scores above 75 indicate an excellent green analysis, scores between 50 and 75 reflect an acceptable green analysis, and scores below 50 denote an inadequate green analysis. The calculation parameters and reference values for deducting penalty points can be found in Table S6 (Supporting Information).

In this context, based on the criteria established by the analytical Eco-Scale, the penalty points assigned to the developed method were as follows: (a) the amount of reagent used per sample (1 point), multiplied by the hazard classification of each reagent (2 points), resulting in a total of 2 penalty points for H_2_O_2_ and an additional 2 points for H_2_SO_4_; (b) energy consumption related to sample preparation using conductive heating (2 points) and sample analysis via ICP-OES (1 point); (c) occupational exposure to vapors and gases generated during acid digestion in an open system, which contributes with 3 penalty points; and (d) waste generation associated with the procedure (5 points), as well as the need for appropriate treatment before disposal (2 points). A detailed breakdown of the penalty points is provided in Table S7 (Supporting Information). Accordingly, the method accumulated a total of 17 penalty points. Subtracting this from the maximum score of 100, the method achieved an analytical Eco-Scale score of 83, reinforcing its suitability as a sustainable alternative for elemental analysis.

## 4 Conclusions

This study contributes to the advancement of elemental analysis in textile materials by developing an efficient, environmentally friendly, and low-cost digestion method specifically adapted for denim fabrics with varying fiber compositions. The methodology enabled the simultaneous determination of seven metals (Al, Co, Cu, Mg, Mn, Ni, and Zn) by ICP OES, achieving satisfactory accuracy and precision following US EPA specifications. A key feature of the proposed approach is the use of conductive heating under mild decomposition conditions, optimized through a design of experiments strategy. This demonstrates the feasibility of replacing more aggressive or costly protocols without compromising analytical performance. The digestion procedure employed low reagent volumes, resulting in minimal hazardous waste generation. From a broader perspective, this methodology not only offers an accessible alternative for laboratories with limited resources but also promotes analytical practices aligned with green chemistry principles. Moreover, the proposed method lays a robust groundwork for future investigations into the elemental composition of denim garments and the potential health risks associated with prolonged exposure to metallic elements. As a whole, it represents a meaningful contribution to strengthening risk assessment strategies within the textile industry.

## Supplementary Material



## Notes

Additional experimental details, materials and methods, certified reference material information, recovery tests results, and penalty points calculations for the analytical Eco-Scale (PDF)

## 1

Universidade Federal Rural de Pernambuco, Unidade Acadêmica de Belo Jardim, Rodovia PE 166, km 5, Euno Andrade da Silva, CEP 55156–580, Belo Jardim, PE, Brazil (I.J.S.d.S.)
