# Supplementary material for: Elemental Composition of Denim Samples via Conductive Heating-Assisted Decomposition with Low Acid Consumption and ICP-OES Detection
Source: ACS Omega. 2025 Sep 2;10(36):41260–9. doi: 10.1021/acsomega.5c04091 (PMC12444594; doi:10.1021/acsomega.5c04091)
Supplement: Supplementary file 1 [file ao5c04091_si_001.pdf]

Elemental Composition of Denim Samples via Conductive Heating-Assisted Decomposition with Low Acid Consumption and ICP-OES Detection

Amanda Laís Barbosa [a], Iago José Santos da Silva [b,1], Briyitte Sofia Salazar Torres [a], Ana Paula Silveira Paim [a,*]

[a] *Universidade Federal de Pernambuco, Centro de Ciências Exatas e da Natureza, Departamento de Química Fundamental, Avenida Jornalista Aníbal Fernandes, s/n, Cidade Universitária, CEP 50740-560, Recife, PE, Brazil.*
[b] *Universidade Federal de Pernambuco, Departamento de Engenharia Civil e Ambiental, Rua Acadêmico Hélio Ramos, s/n, Cidade Universitária, CEP 50740-467, Recife, PE, Brazil.*

[1] Iago J. S. da Silva (current address): *Universidade Federal Rural de Pernambuco, Unidade Acadêmica de Belo Jardim, Rodovia PE 166, km 5, Euno Andrade da Silva, CEP 55156-580, Belo Jardim, PE, Brazil.*

Supporting Information

Table S1 – Coding of the denim samples acquired: description, origin, composition, and color.

Code	Description	Origin	Composition [a]	Color
T1	Moderate stretch fabric	Recife	97% CO; 3% EA	Green
T2	Moderate stretch fabric	Recife	97% CO; 3% EA	Dark blue
T3	Non-elastic fabric	Recife	98% CO; 2% EA	Grey
T4	High elasticity fabric	Recife	96% CO; 4% EA	Dark blue
T5	Non-elastic fabric	Recife	100% CO	Medium blue
T6	Non-elastic fabric	Recife	100% CO	Dark blue
T7	Children's jeans	Recife	82% CO; 16% PL; 2% EA	Medium blue
T8	Children's shorts	Caruaru	98% CO; 2% EA	Dark blue
T9	Children's shorts	Caruaru	97% CO; 3% EA	Light blue
T10	Children's shorts	Caruaru	97% CO; 3% EA	Dark blue
T11	Children's women's shorts	Caruaru	77% CO; 20% PL; 3% EA	Medium blue
T12	Children's women's shorts	Caruaru	77% CO; 20% PL; 3% EA	Dark blue
T13	Children's short skirt	Caruaru	100% CO	Light blue
T14	Children's short skirt	Caruaru	100% CO	Dark blue
T15	Children's short skirt	Caruaru	100% CO	Light blue
T16	Female skirt	Caruaru	100% CO	Light blue
T17	Women's shorts	Caruaru	100% CO	Black
T18	Children's shorts	Caruaru	100% CO	Medium blue
T19	Children's shorts	Caruaru	100% CO	Medium blue
T20	Children's shorts	Caruaru	98% CO; 2% EA	Light blue
T21	Children's shorts	Caruaru	98% CO; 2% EA	Medium blue
T22	Children's shorts	Caruaru	100% CO	Dark blue
T23	Women's shorts	Caruaru	98% CO; 2% EA	Dark blue
T24	Female pants	Caruaru	75% CO; 22% PL; 3% EA	Light blue
T25	Female pants	Caruaru	75% CO; 22% PL; 3% EA	Dark blue
T26	Children's shorts	Caruaru	98% CO; 2% EA	Dark blue
T27	Children's shorts	Caruaru	100% CO	Dark blue
T28	Children's shorts	Caruaru	100% CO	Dark blue
T29	Children's skirts	Caruaru	100% CO	Dark blue

[a] Fiber composition: cotton (CO), elastane (EA), and polyester (PL).

Table S2 – Reference values for the mass fraction (on a dry basis) attributed to the elements determined in the certified reference material (CRM-Agro E1001a) and expanded uncertainty (U_{MR}), following the information presented in the certificate of analysis [a].

Element	Average concentration ± U_{MR} (mg kg^{-1}) [b]	U_{MR} (%) [c]
As	1.69 ± 0.70	41
Cr	3.30 ± 1.66	50
Pb	4.0 ± 1.8	45
Cd	19.9 ± 5.1	26
Ca	4.37 ± 0.58	13
Mg	2.95 ± 0.44	15
Na	0.19 ± 0.10	53
Fe	91 ± 13	14
Cu	4.0 ± 0.7	17
Zn	9.9 ± 1.6	16
K	12.0 ± 2.4	20
Mn	76.0 ± 18.5	24
P	0.65 ± 0.19	29

[a] The certified reference material was acquired from Empresa Brasileira de Pesquisa Agropecuária (EMBRAPA), a Brazilian public institution, linked to the Ministério da Agricultura e Pecuária (MAPA). All values presented in the table were obtained from the Analysis Certificate provided by the institution along with the CRM. The reference material was produced from a forage sample (*Brachiaria brizantha* Stapf. cv. Marandu) and coded as CRM-Agro E1001a.

[b] 95% confidence interval.

[c] Number of accepted participating laboratory results that were used to calculate consensus values.

Table S3 – Visual aspect observed from the application of different acid solutions in the digestion of denim samples.

Acid Solution	Visual aspect	References [a]
H_2O_2/HNO_3	Colored solution and occurrence of solid residue in small to large quantities	[7-9]
HNO_3/H_2SO_4	Colored solution and partially decomposed sample pieces	[22-23]
H_2SO_4/H_2O_2	Absence of particulate material and coloration	[24]

[a] The original volumes suggested by the references were adjusted to a mass of 200 mg of denim.

Table S4 – Conditions established through experimental design study for the digestion of denim samples (200 mg): first (I) and second step (II).

Reaction mixture	(I) 2 mL of H_2SO_4 (98%) + 1 mL of H_2O_2 (30%)
	(II) 4 mL of H_2O_2 (30%)
Heating ramp	(I) The system is heated from room temperature to 120 °C
	(II) Temperature is maintained at 120 °C for 60 minutes
Final volume	30 mL

Table S5 – Recovery tests at two concentration levels.

Levels (mg L^{-1})		T3		T7		T24		T29	
		Rec (%)	RSD (%)	Rec (%)	RSD (%)	Rec (%)	RSD (%)	Rec (%)	RSD (%)
Al	0.4	134	0.7	121	10.3	107	3.1	102	0.6
	0.8	121	10.3	109	7.9	85.1	1.9	93.9	5.1
Co	0.4	92.2	4.8	92.3	5.3	77.5	4.9	75.7	0.3
	0.8	92.3	5.3	87.7	17.7	74.1	5.1	77.1	3.6
Cr	0.4	85.3	2.5	85.3	6.8	72.9	0.5	69.8	2.3
	0.8	85.3	6.8	84.2	19.8	70.3	4.8	73.6	8.0
Cu	0.4	77.4	0.1	85.2	6.8	75.9	3.6	82.3	6.9
	0.8	85.2	6.8	82.1	4.4	73.2	4.2	77.1	2.1
Fe	0.4	140	0.2	194	32.3	174	14.3	172	5.6
	0.8	185	12.0	146	4.5	160	11.2	174	4.5
Mg	2.0	116	1.7	80.5	12.7	86.8	8.4	96.9	5.6
	4.0	80.5	12.7	85.2	13.6	88.1	7.3	93.5	3.0
Mn	0.4	83.9	0.8	91.6	4.3	76.9	4.7	73.3	7.9
	0.8	91.6	4.3	90.3	16.5	75.3	12.0	81.0	1.5
Ni	0.4	96.3	1.1	94.4	3.0	82.3	2.8	78.8	0.6
	0.8	94.4	3.0	91.5	18.4	78.5	7.8	84.1	1.3
Zn	0.4	41.1	0.2	86.4	6.8	93.2	19.8	103	9.6
	0.8	86.4	6.8	88.9	13.8	84.7	9.3	93.8	0.5

[a] Mean value ± standard deviation (n = 3).

Table S6 – The penalty points (PPs) to calculate the analytical Eco-Scale

		Sub-total PP	Total PP
**Amount of Reagent**	< 10 mL (g)	1	Amount × Hazard
	10 – 100 mL (g)	2	
	> 100 mL (g)	3	
**Hazard of Reagent**	None	0	Amount × Hazard
	Less severe hazard	1	
	More severe hazard	2	
**Energy Consumption of Instruments**	≤ 0.1 kWh per sample		0
	≤ 1.5 kWh per sample		1
	> 1.5 kWh per sample		2
**Occupational Hazard**	Analytical process hermetization		0
	Emission of vapors and gases to the air		3
**Amount of Waste**	None		0
	< 1 mL (g)		1
	1–10 mL (g)		3
	> 10 mL (g)		5
**Waste Treatment**	Recycling		0
	Degradation		1
	Passivation		2
	No treatment		3

[a] PPs are assigned to each of the hazard categories posed by a reagent. Each reagent can have more than one hazard category; thus, the sub-total PP value for a single reagent may be greater than 2.
[b] Gałuszka, A.; Konieczka, P.; Migaszewski, Z. M.; Namiesnik, J. **Analytical Eco-Scale for assessing the greenness of analytical procedures**. *Trends in Analytical Chemistry*, **2012**, 37, 61–72. doi: 10.1016/j.trac. 2012.03.013.

Table S7 – The penalty points (PPs) for elemental composition of denim samples via conductive heating-assisted diluted acid decomposition and ICP-OES detection.

**Reagents**	**Penalty points**
H_2O_2 (More severe hazard and 5 mL per sample)	2
H_2SO_4 (More severe hazard and 2 mL per sample)	2
	Σ 4
**Instruments**	**Penalty points**
Digester block (more than 1.5 kWh per sample)	2
ICP OES (up to 1.5 kWh per sample)	1
Occupational hazard (emission of vapors and gases)	3
Waste generation (more than 10 mL per sample)	5
Waste treatment (acid-base neutralization)	2
	Σ 13
Total penalty points: 17	
**Analytical Eco-Scale total score: 83**	
